# Spontaneous splenic rupture in pregnancy

**DOI:** 10.11604/pamj.2015.21.312.6878

**Published:** 2015-08-28

**Authors:** Adil Elghanmi, Jou Mohamed, Samira Khabouz

**Affiliations:** 1 Department de Gynecology and Obstetrics 1, Maternité Souissi, Chu Ibn Sina, Rabat, Morocco

**Keywords:** Splenic rupture, pregnancy, hypovolemic shock

## Abstract

Splenic rupture during pregnancy is a rare and can frequently be a misdiagnosed pathology. This rupture is associated with a high maternal and fetal mortality rate. A 26 years old Moroccan woman para II gravida II presented at the third stage of pregnancy with acute onset of severe abdominal pain. She developed immediately a hypovolemic shock. After both a physical and sonographical exam, it was revealed that it was due to a massive hemoperitoneum. Therefore, an emergent laparotomy and cesarean delivery with abdominal exploration were performed; also, an active bleeding was identified at the splenic hilum consistent with splenic rupture. Through this case report, we want to raise awareness of this surgical emergency that requires immediate recognition because any delay can lead to catastrophic consequences

## Introduction

A spontaneous rupture of the spleen is a rare entity. It often occurs in pre-existing pathology of the spleen, such as a splenic artery aneurysm or thalassemia, or infectious etiologies such as malaria, typhoid, or infectious mononucleosis, but most commonly after a trauma [1]. The clinical feature may mimic an ectopic or an abdominal pregnancy, a uterine rupture or an abruption of the placenta. The delay in recognition of this rupture can lead to catastrophic consequences for both the mother and the fetus.

## Patient and observation

A 26 years old Moroccan woman in the category of para II gravida II was admitted to the obstetrical emergency unit, after experiencing upper abdominal pain, vertigo and general weakness at home. This patient had no significant medical history prior to the onset of pregnancy. At admission, she was pale and had signs of hemorrhagic shock. Also, she had a high pulse of 140 beat per minute and a blood pressure of 60/40 mmHg. To stabilize the situation, immediately, intravenous access was established and plasma expenders and oxygen were given to this patient. Furthermore, this patient had no contraction, the uterus was tender and the cervix wasn't dilated.

Ultrasonographic exam revealed a non viable intra uterine pregnancy at 35 week of gestation and an extensive intra abdominal fluid. Emergent laparotomy confirmed the important hemoperitoneum of approximately 3 liters. A low hysterotomy was performed, amniotic fluid noted clear and a dead fetus was delivered. There was no uterine cause of the bleeding. An upper midline laparotomy was performed. Active bleeding was identified at the splenic hilum consistent with splenic rupture. A splenectomy was performed by a general surgeon. After temporary stabilization of blood pressure by infusion and blood transfusion, disseminated vascular coagulation set in. Despite all of our attempts, this patient died of a sudden cardiac arrest, about 7 hours after her admission.

## Discussion

Spontaneous rupture of the spleen in pregnancy is a rare clinical event [2]. A spontaneous rupture of a normal spleen is a rupture in the absence of antecedent trauma, a systemic disease that can affect the spleen, a spleen cyst at the time of exploration, and the spleen parenchyma, vasculature, and capsule should be normal macroscopically and histologically [3]. The etiology of spontaneous rupture of a normal spleen in pregnancy remains speculative at best [1]. It has been suggested that splenic enlargement, increased blood volume and reduced peritoneum cavity during pregnancy could be implicated in the pathogenesis of splenic rupture [3]. Circulating hormones such as estrogen and progesterone cause structural changes to the spleen that may increase the risk of splenic rupture during pregnancy even after minor trauma [1].

Splenic rupture is more common in the third trimester, but some cases of rupture occurred in the post partum period [2, 4]. The splenic rupture during pregnancy is difficult to diagnose because it shares signs and symptoms with other conditions such as uterine rupture and abruption of the placentae. The standard of care for patient with spontaneous splenic rupture remains splenectomy [3]. Maternal death is commonly due to massive hemorrhage and accompanying hemorrhagic shock and consumptive coagulopathy. Maternal hemodynamic decompensation will lead to an acute decrease in uteroplacental perfusion, resulting in “fetal distress” and, ultimately, fetal demise [1].

## Conclusion

Splenic rupture in pregnancy is a life threatening situation. It should be considered as a diagnosis of hemoperitoneum without a uterine explication. Emergent laparotomy and splenectomy before the setting of collapse and disseminated vascular coagulation are the essential keys to enhance maternal and increase the chances of fetal survival.
